# Association of FIB‐4, APRI, and HellenicSCORE II With Chronic Kidney Disease in Patients With Type 2 Diabetes Mellitus

**DOI:** 10.1155/jdr/8029869

**Published:** 2026-08-28

**Authors:** Katerina Stefanaki, Dimitrios S. Karagiannakis, Maria Manolakopoulou, Stefania Magkou, Paraskevi Kazakou, Panagiota Zacharaki, Melpomeni Peppa, Evanthia Kassi, Theodora Psaltopoulou, Stavroula A. Paschou

**Affiliations:** ^1^ Endocrine Unit and Diabetes Center, Department of Clinical Therapeutics, Alexandra Hospital, Medical School, National and Kapodistrian University of Athens, Athens, Greece, uoa.gr; ^2^ 4th Department of Internal Medicine, “Attikon” University Hospital, School of Medicine, National and Kapodistrian University of Athens, Athens, Greece, uoa.gr; ^3^ Department of Clinical Therapeutics, Alexandra Hospital, Medical School, National and Kapodistrian University of Athens, Athens, Greece, uoa.gr; ^4^ Medical School, National and Kapodistrian University of Athens, Athens, Greece, uoa.gr; ^5^ Endocrine Unit, 2nd Department of Internal Medicine-Propaedeutic, Research Institute and Diabetes Center, Medical School, National and Kapodistrian University of Athens, Athens, Greece, uoa.gr; ^6^ 1st Department of Propaedeutic Internal Medicine, Medical School, National and Kapodistrian University of Athens, Athens, Greece, uoa.gr

**Keywords:** APRI, chronic kidney disease, diabetes mellitus, FIB-4, HellenicSCORE II, MASLD

## Abstract

**Background:**

Metabolic dysfunction–associated steatotic liver disease (MASLD) commonly affects patients with Type 2 diabetes mellitus (T2DM). Fibrosis‐4 (FIB‐4) has been linked to chronic kidney disease (CKD) in patients with MASLD; however, its association with CKD in broader, not preselected for MASLD, T2DM populations remains unclear.

**Aim:**

To investigate the association of FIB‐4 with CKD in unselected patients with T2DM and compare its discriminatory ability with that of HellenicSCORE II and aspartate aminotransferase‐to‐platelet ratio index (APRI).

**Methods:**

We retrospectively analyzed data from consecutive patients with T2DM. Characteristics and baseline laboratory results were recorded.

**Results:**

A total of 214 patients (mean age 67.6 ± 10.3 years) were enrolled, including 158 (73.8%) males and 56 (26.2%) females. Ninety‐six (44.8%) had normal kidney function, and 54 (25.2%) had CKD at enrollment. Those with CKD were significantly older (*p* = 0.002) and had higher scores on the HellenicSCORE II (*p* = 0.004), APRI (*p* = 0.001), and FIB‐4 (*p* < 0.001). Additionally, hypertension and cardiovascular disease were more common in the CKD than in the non‐CKD group (*p* < 0.001 for both). In the multivariate analysis, sex (OR: 0.13, 95% CI: 0.03–0.6; *p* = 0.008) and FIB‐4 (OR: 10.4, 95% CI: 2–54.1; *p* = 0.005) were independently associated with CKD. Among the evaluated scores, FIB‐4 demonstrated the highest numerical AUROC for identifying CKD (0.769; 95% CI: 0.668–0.869), compared with APRI (0.668; 95% CI: 0.558–0.778) and HellenicSCORE II (0.651; 95% CI: 0.544–0.757).

**Conclusions:**

FIB‐4 was independently associated with the presence of CKD in patients with T2DM and demonstrated the highest numerical AUROC among the evaluated scores.

## 1. Introduction

The incidence of Type 2 diabetes mellitus (T2DM) has risen globally in recent years [1]. According to the International Diabetes Federation, over half a billion adults around the world are currently affected by diabetes, with projections indicating further growth in the prevalence of the condition in the near future [1, 2]. T2DM is linked to significant complications, which contribute to increased overall morbidity and mortality [3, 4]. Chronic kidney disease (CKD) is a prevalent complication, occurring in nearly 30% of individuals with T2DM, and it is linked to a higher risk of cardiovascular disease (CVD) [5] and unfavorable outcomes [6–8].

Metabolic dysfunction–associated steatotic liver disease (MASLD) is often present in patients with T2DM, sharing similar pathophysiological mechanisms [9]. It covers a range of conditions from simple steatosis to steatohepatitis, severe liver fibrosis, and cirrhosis [10]. Beyond liver disease, MASLD is also linked to extrahepatic disorders, such as CVD [11], CKD [12], and extrahepatic cancers [13]. Liver fibrosis is the strongest predictor of liver‐related outcomes [14] and has been consistently linked with extrahepatic complications, including CKD [15].

Fibrosis‐4 (FIB‐4) has been recommended as the initial noninvasive test in a two‐step approach to evaluate significant fibrosis in patients with MASLD in the general population, given its high negative predictive value (NPV) of approximately 90%–95% [16]. In addition, FIB‐4 appears to reflect systemic inflammation and metabolic state, as research has linked FIB‐4 levels with CVD and extrahepatic cancers [17, 18].

Regarding CKD, studies have shown an association between FIB‐4 levels and the presence or progression of CKD across diverse populations and clinical settings [19, 20]. Particularly for patients with T2DM, elevated FIB‐4 values have shown an association with CKD and adverse renal outcomes in several high‐risk groups, including those with concurrent MASLD, older age, or pre‐existing kidney issues [21–23]. However, it remains unclear whether FIB‐4 is associated with CKD in routine‐care populations of patients with T2DM who are not preselected for kidney disease or MASLD.

In addition to FIB‐4, other clinical scores may capture features associated with kidney dysfunction. Aspartate aminotransferase (AST)‐to‐platelet ratio index (APRI) is a simple, routinely available noninvasive marker, and previous studies have reported associations between APRI and CKD, although the available evidence is considerably more limited than that for FIB‐4 [20]. Beyond liver‐related indices, cardiovascular assessment may also be relevant to kidney dysfunction, given the substantial overlap between the clinical and metabolic factors associated with cardiovascular and kidney disease. Indeed, individuals with a higher estimated cardiovascular risk have been shown to exhibit an increased incidence of CKD [24]. In this context, HellenicSCORE II, a cardiovascular risk assessment model specifically recalibrated for the Greek population [25], incorporates several demographic and clinical variables that are also associated with kidney dysfunction. We hypothesized that HellenicSCORE II might be associated with CKD in patients with T2DM and included it as a cardiovascular‐based comparator to the liver‐related indices.

Therefore, the present study is aimed at evaluating the associations of FIB‐4, APRI, and HellenicSCORE II with the presence of CKD in a consecutive, real‐world cohort of patients with T2DM, irrespective of MASLD status or known renal dysfunction. We further compared the ability of these readily available scores to discriminate between patients with and without CKD.

## 2. Materials and Methods

### 2.1. Patients

We retrospectively examined data from consecutive patients aged ≥ 18 years with a diagnosis of T2DM for at least 1 year who attended our diabetes center between September 1, 2023, and August 31, 2024. Patients were not preselected based on kidney disease or MASLD. Those with known chronic liver diseases, those with excessive alcohol consumption [10], or those who had been exposed to potentially hepatotoxic drugs were excluded from the study.

### 2.2. Methods

For all patients, we collected epidemiological and somatometric data and performed blood tests. We documented the patient′s history of hypertension and CVD. Furthermore, we recorded the duration of T2DM, the antidiabetic medications prescribed, and the duration of use from treatment initiation until recruitment. The glomerular filtration rate (GFR) was calculated using the CKD‐EPI 2021 formula [26]. A diagnosis of CKD was made using a CKD‐EPI score of 60 mL/min/1.73 m^2^ or less for more than 3 months [27]. Additionally, we assessed the HellenicSCORE II [25], FIB‐4 [28], and APRI [28] at enrollment. FIB‐4 was calculated as age  ×  AST/[platelet count  ×  √alanine aminotransferase (ALT)]. APRI was calculated as [(AST/upper limit of normal AST)/platelet count] × 100. The HellenicSCORE II was calculated according to the original model by Panagiotakos et al. [25].

### 2.3. Ethics

The protocol was consistent with the Helsinki Declaration for Human Studies, as updated in 1983, and received approval from the institution′s ethics committee. Written informed consent was obtained from all participants for the anonymous use of their data.

### 2.4. Statistical Analysis

Statistical analysis was performed using SPSS Version 30 software (SPSS Inc., Chicago, Illinois, United States). Continuous variables were evaluated for normality through the Kolmogorov–Smirnov and Shapiro–Wilk tests. They were reported as either the mean ± standard deviation (SD) or the median (interquartile range [IQR]), depending on the data distribution. Categorical variables were expressed as the number of cases with their corresponding percentages. Quantitative variables were analyzed using Student′s *t*‐test for normally distributed data and the Mann–Whitney *U* test for those that were not normally distributed. Qualitative variables were compared employing the corrected chi‐squared test or the two‐sided Fisher′s exact test, where applicable. The associations between parameters were assessed with Spearman′s correlation coefficient. Variables significant in the univariate analysis were included in a forward stepwise multivariate logistic regression to identify factors associated with CKD; odds ratios (ORs) and their 95% confidence intervals (CIs) were reported. Receiver operating characteristic (ROC) analysis was conducted to evaluate FIB‐4′s ability to distinguish between patients with and without CKD at enrollment, and the optimal cutoff was determined by maximizing the Youden index [29]. All tests were two‐sided, and *p* values below 0.05 were deemed statistically significant.

## 3. Results

A total of 214 consecutive patients were enrolled. One hundred fifty‐eight (73.8%) patients were male, and 56 (26.2%) were female. One hundred eighty‐six (86.9%) were Greek, and 28 (13.1%) were of non‐Greek nationality. The mean age was 67.7 ± 10.3 years. The mean duration of T2DM was 10 ± 8.1 years. At baseline, 96 (44.8%) patients had normal kidney function, and 54 (25.2%) had CKD. Of the total, 64 patients (29.9%) had missing data, preventing the calculation of their GFR. Comparison between patients with available and unavailable GFR data did not reveal significant differences in age, sex distribution, diabetes duration, body mass index (BMI), APRI, FIB‐4, or hypertension prevalence (data not shown). Patients′ demographics are depicted in Table 1.

**Table 1:** Patients′ characteristics at enrollment.

Variable	Values
Age (years)	67.7 ± 10.3
Sex (males/females)	158 (73.8%)/56 (26.2%)
Nationality (Greek/foreign)	186 (86.9%)/28 (13.1%)
BMI (kg/m^2^)	31.3 ± 5.3
Diabetes duration (years)	10 ± 8.1
GLP‐1 analog duration (years)	1.7 ± 1.4
SGLT‐2 inhibitor duration (years)	1.8 ± 1.7
Cr (mg/dL)	0.99 ± 0.35
AST (U/L)	20.4 ± 7.3
ALT (U/L)	24 ± 13.4
*γ*‐GT (U/L)	35 ± 63.3
ALP (IU/L)	85.5 ± 34.9
LDL (mg/dL)	90.1 ± 41.1
HDL (mg/dL)	45 ± 13.2
Tg (mg/dL)	171.1 ± 108.8
Platelet counts (×10^3^/*μ*L)	271.4 ± 108.3
Glycosylated hemoglobin (%)	7.5 ± 1.8
TSH (*μ*U/mL)	2.5 ± 1.7
APRI	0.21 ± 0.14
FIB‐4	1.2 ± 0.6
HellenicSCORE II	5.6 ± 4

*Note:* Qualitative variables are reported as counts (*n*) and percentages (%). Continuous variables are expressed as mean ± standard deviation (SD).

Abbreviations: *γ*‐GT, gamma‐glutamyl transferase; ALP, alkaline phosphatase; ALT, alanine aminotransferase; APRI, aspartate aminotransferase‐to‐platelet ratio index; AST, aspartate aminotransferase; BMI, body mass index; Cr, creatinine; FIB‐4, Fibrosis‐4 index; GLP‐1, Glucagon‐Like Peptide‐1; HDL, high‐density lipoprotein; LDL, low‐density lipoprotein; SGLT‐2, Sodium–Glucose Transporter‐2; Tg, triglyceride; TSH, thyroid‐stimulating hormone.

**Figure 1:**
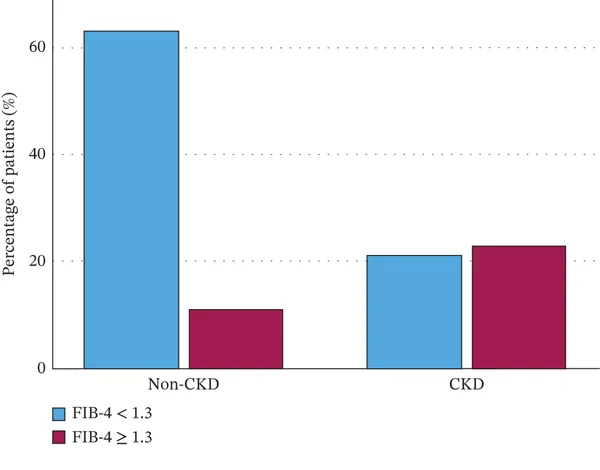
Differences in CKD prevalence according to FIB‐4 levels.

**Figure 2:**
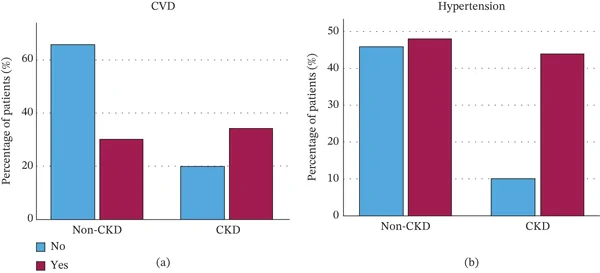
CKD prevalence according to history of (a) cardiovascular disease or (b) the presence of hypertension.

Individuals with CKD were notably older (*p* = 0.002), had a higher HellenicSCORE II (*p* = 0.004), exhibited a lower platelet count (*p* < 0.001), presented elevated AST levels (*p* = 0.031), had a greater APRI score (*p* = 0.001), and showed a higher FIB‐4 score (*p* < 0.001) compared with those without CKD (Table 2). Furthermore, a larger number of patients with CKD had FIB − 4 ≥ 1.3 (*p* < 0.001) (Figure 1), CVD (*p* < 0.001), and hypertension (*p* < 0.001) compared with patients without CKD (Figure 2).

**Table 2:** Differences between patients with and without CKD.

Variable	Non‐CKD (*n* = 96)	CKD (*n* = 54)	*p* value
Age (years)	64.5 ± 10.2	70.2 ± 7.8	0.002
BMI (kg/m^2^)	30.8 ± 5.4	32.7 ± 5.7	0.062
Diabetes duration (years)	10.1 ± 8.2	10.6 ± 8.2	0.795
Years of treatment with GLP‐1 analogs	1.6 ± 1.2	1.7 ± 1.8	0.657
Years of treatment with SGLT‐2 inhibitors	1.7 ± 1.4	1.8 ± 2	0.532
AST (U/L)	18.7 ± 5.3	22.5 ± 9.7	0.031
ALT (U/L)	22.7 ± 10.9	26.7 ± 15.9	0.329
*γ*‐GT (U/L)	27.1 ± 16.9	51.9 ± 118.9	0.424
ALP (IU/L)	73.2 ± 35	95.8 ± 34.9	0.797
LDL^a^ (mg/dL)	97.4 ± 44.2	74.6 ± 34.8	0.003
HDL^a^ (mg/dL)	45.2 ± 13	43.7 ± 11	0.607
Tg^a^ (mg/dL)	174.6 ± 129	162.6 ± 70.4	0.489
Platelet count (×10^3^/*μ*L)	294.8 ± 105.9	240.2 ± 108.7	< 0.001
Glycosylated hemoglobin (%)	7.3 ± 1.9	7.5 ± 1.2	0.035
TSH (*μ*U/mL)	2.5 ± 1.1	2.6 ± 2.3	0.065
APRI	0.18 ± 0.08	0.27 ± 0.21	0.001
FIB‐4	0.94 ± 0.38	1.48 ± 0.76	< 0.001
HellenicSCORE II	4.7 ± 3.6	6.5 ± 3.5	0.004

*Note:* Qualitative variables are reported as counts (*n*) and percentages (%). Continuous variables are expressed as mean ± standard deviation (SD).

Abbreviations: *γ*‐GT, gamma‐glutamyl transferase; ALT, alanine aminotransferase; ALP, alkaline phosphatase; APRI, aspartate aminotransferase‐to‐platelet ratio index; AST, aspartate aminotransferase; BMI, body mass index; CKD, chronic kidney disease; FIB‐4, Fibrosis‐4 index; GLP‐1, Glucagon‐Like Peptide‐1; HDL, high‐density lipoprotein; LDL, low‐density lipoprotein; SGLT‐2, Sodium–Glucose Transporter‐2; Tg, triglyceride; TSH, thyroid‐stimulating hormone.

^a^LDL, HDL, and Tg levels are after treatment with statins. Statins were administered to all our patients.

Baseline Cr levels were found to correlate positively with age (rho = 0.303; *p* < 0.001), HellenicSCORE II (rho = 0.256; *p* = 0.003), APRI (rho = 0.21; *p* = 0.022), and FIB‐4 (rho = 0.355; *p* < 0.001) and negatively with platelet count (rho = −0.288; *p* = 0.001). Cr levels were not statistically correlated with diabetes duration, BMI, serum levels of AST, ALT, gamma‐glutamyl transferase (*γ*‐GT), alkaline phosphatase (ALP), high‐density lipoprotein (HDL), low‐density lipoprotein (LDL), triglyceride (Tg), thyroid‐stimulating hormone (TSH), glycosylated hemoglobin (HbA1c), years of taking Sodium–Glucose Transporter‐2 (SGLT‐2) inhibitors, or years of taking Glucagon‐Like Peptide‐1 (GLP‐1) analogs.

### 3.1. Univariate and Multivariate Analyses

In the univariate analysis, age, sex, nationality, SGLT‐2 inhibitor administration, HellenicSCORE II, AST levels, platelet count, APRI, FIB‐4 levels, FIB − 4 ≥ 1.3, hypertension, and CVD were associated with CKD at the time of recruitment (Table 3). In the multivariate analysis, after adjusting for age, AST, and platelet count to avoid multicollinearity with FIB‐4, only sex and FIB‐4, either as a continuous variable or as a categorical variable (FIB − 4 ≥ 1.3 vs. < 1.3), were independently associated with CKD (Table 4).

**Table 3:** Univariate analysis of factors associated with CKD presence.

Variable	OR	95% CI	*p* value
Age (years)	1.07	1.03–1.1	< 0.001
Sex (male vs. female)	0.18	0.07–0.45	< 0.001
Nationality (Greek vs. other)	0.17	0.04–0.75	0.019
Treatment with SGLT‐2 inhibitors	2.1	1.03–4.4	0.042
HellenicSCORE II	1.15	1.03–1.3	0.009
ASTT (U/L)	1.08	1.02–1.14	0.006
Platelets	1.0	1.0–1.0	0.01
APRI	577.8	9.5–35017.9	0.002
FIB‐4 levels	6.7	2.8–16.3	< 0.001
FIB‐4 (≥ 1.3 vs. < 1.3)	6.3	2.6–15	< 0.001
Hypertension	4.2	1.9–9.4	< 0.001
CVD	3.7	1.9–7.5	< 0.001

Abbreviations: APRI, aspartate aminotransferase‐to‐platelet ratio index; AST, aspartate aminotransferase; CI, confidence interval; CVD, cardiovascular disease; FIB‐4, Fibrosis‐4 index; OR, odds ratio; SGLT‐2, Sodium‐–Glucose Transporter‐2.

**Table 4:** Multivariate analysis of the factors independently associated with CKD presence.

Variable	OR	95% CI	*p* value
(a) Model adjusted for sex, nationality, treatment with SGLT‐2 inhibitors, HellenicSCORE II, APRI, FIB‐4 as a continuous variable, hypertension, and CVD (excluding age, AST, and platelets, which are components of the FIB‐4 index)			
Sex (male vs. female)	0.13	0.03–0.6	0.008
FIB‐4 levels	10.4	2–54.1	0.005
(b) Model adjusted for sex, nationality, treatment with SGLT‐2 inhibitors, HellenicSCORE II, APRI, FIB‐4 as a qualitative variable, hypertension, and CVD (excluding age, AST, and platelets, which are components of the FIB‐4 index)			
Sex (male vs. female)	0.1	0.02–0.5	0.005
FIB − 4 ≥ 1.3 vs. < 1.3	7.5	1.8–30.9	0.00

Abbreviations: APRI, aspartate aminotransferase‐to‐platelet ratio index; AST, aspartate aminotransferase; CI, confidence interval; CVD, cardiovascular disease; FIB‐4, Fibrosis‐4 index; OR, odds ratio; SGLT‐2, Sodium–Glucose Transporter‐2.

To further explore whether the association between FIB‐4 and CKD was predominantly driven by age, an additional multivariable analysis was performed including age, FIB‐4, and the other variables associated with CKD in the univariate analyses. FIB‐4 remained significantly associated with CKD (OR 21.9, 95% CI 2.03–236.8; *p* = 0.011), whereas age was no longer significantly associated with CKD (OR 0.95, 95% CI 0.84–1.07; *p* = 0.370).

### 3.2. FIB‐4 in Identifying the Presence of CKD

**Figure 3:**
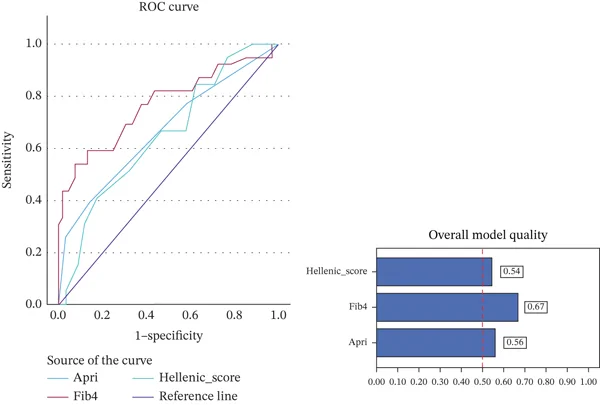
AUROCs of FIB‐4, APRI, and HellenicSCORE II for discriminating between patients with and without CKD.

The AUROC for the FIB‐4 score in identifying CKD was 0.769 (95% CI: 0.668, 0.869). The optimal FIB‐4 cutoff was 1.54, with 54% sensitivity and 93% specificity. APRI score yielded an AUROC of 0.668 (95% CI: 0.558, 0.778), and the HellenicSCORE II yielded an AUROC of 0.651 (95% CI: 0.544, 0.757) (Figure 3).

## 4. Discussion

In this study, the association between FIB‐4 and the presence of CKD was investigated retrospectively in a consecutive, real‐world cohort of patients with T2DM, non‐preselected for MASLD or known renal dysfunction, treated at a tertiary diabetes center. Approximately 25% of our patients had a GFR ≤ 60 mL/min/1.73 m^2^ at recruitment. Those patients were older and had higher FIB‐4, APRI, and HellenicSCORE II values.

Consistent with these findings, serum creatinine levels were positively correlated with both FIB‐4 and HellenicSCORE II, and CVD was more frequent among patients with CKD, further illustrating the close relationship between renal and cardiovascular dysfunction in patients with T2DM. Moreover, patients with CKD were more likely to have FIB‐4 values ≥ 1.3. Importantly, however, the association between higher FIB‐4 values and CKD should not necessarily be interpreted as reflecting a greater liver fibrotic burden. Given that FIB‐4 incorporates age, AST, ALT, and platelet count, its elevation in patients with CKD may partly reflect age‐related and kidney dysfunction–associated alterations in these parameters. CKD itself may affect aminotransferase levels and platelet count through metabolic and inflammatory alterations, uremia‐related hematologic changes, and changes in muscle mass. Thus, in this setting, FIB‐4 may capture systemic alterations associated with CKD, either in addition to or independently of underlying liver fibrosis.

The association between FIB‐4 and CKD was further supported by the univariate analysis, in which FIB‐4, analyzed both as a continuous variable and using the ≥ 1.3 cut‐off, was associated with a higher prevalence of CKD. Besides FIB‐4, older age, female sex, foreign nationality, history of CVD, hypertension, AST levels, platelet count, APRI, and treatment with SGLT‐2 inhibitors were also associated with CKD. Although APRI demonstrated a significant association with CKD, the wide CI indicates limited precision and potential model instability, which may be attributable to the relatively small number of patients with CKD. Regarding SGLT‐2 inhibitors, the observed association with CKD likely reflects confounding by indication, as these agents are preferentially prescribed to patients with established or high‐risk kidney disease [30]. In the multivariate analyses, only FIB‐4, either continuously or categorically (≥ 1.3 vs. < 1.3), and sex remained independently associated with CKD. Given that age is incorporated into the FIB‐4 formula and is an important determinant of CKD, we further explored whether the observed association between FIB‐4 and CKD was predominantly driven by age. Although age was significantly associated with CKD in univariate analysis, AST and platelet count, two nonage components of FIB‐4, were also significantly associated with CKD. Furthermore, in an extended multivariable model that included age, FIB‐4, and other variables associated with CKD in univariate analyses, FIB‐4 remained significantly associated with CKD, whereas age was not. These findings suggest that the association between FIB‐4 and CKD is unlikely to be explained solely by the age component of FIB‐4. Nevertheless, because age is mathematically incorporated into FIB‐4, its contribution cannot be completely disentangled, and residual age‐related confounding cannot be excluded.

The relationship between CKD and FIB‐4, as demonstrated in our study, has already been well documented in individuals with MASLD [19]. However, in patients with T2DM, evidence for an association between FIB‐4 and CKD has primarily been derived from selected high‐risk subgroups with multiple comorbidities [21–23]. The present study extends these observations by demonstrating an association between FIB‐4 and CKD in a consecutive, routine‐care cohort of patients with T2DM, not preselected for MASLD or known renal dysfunction, providing evidence that more closely reflects everyday clinical practice in an outpatient diabetes department.

The association between FIB‐4 and CKD should also be considered within the broader cardio–renal–metabolic context of T2DM. T2DM, CKD, and MASLD frequently coexist and share several pathophysiological mechanisms, including insulin resistance, chronic low‐grade inflammation, oxidative stress, adipose tissue dysfunction, and endothelial dysfunction [31]. These interconnected mechanisms may contribute not only to kidney and liver disease but also to the increased cardiovascular burden observed in patients with metabolic dysfunction. Indeed, MASLD, particularly in the presence of advanced liver fibrosis, has been associated with an increased risk of CVD, including coronary artery disease, heart failure, atrial fibrillation, and cerebrovascular disease [32]. Therefore, although FIB‐4 was originally developed as a noninvasive marker of hepatic fibrosis, its association with CKD may not necessarily reflect hepatic fibrosis alone but may partly capture the broader age‐related and cardiometabolic abnormalities accompanying T2DM and CKD. This interpretation is particularly relevant in our cohort, in which MASLD and hepatic fibrosis were not systematically assessed by imaging or elastography.

FIB‐4 demonstrated the highest numerical AUROC among the three evaluated scores. Specifically, the AUROC of FIB‐4 for detecting CKD was 0.769, whereas those of APRI and HellenicSCORE II were 0.668 and 0.651, respectively. However, as formal pairwise comparisons between ROC curves were not performed, these differences should not be interpreted as evidence of statistical superiority.

From a clinical perspective, FIB‐4 should not be considered a substitute for established CKD screening based on eGFR and urinary albumin‐to‐creatinine ratio (UACR), nor should an elevated FIB‐4 alone constitute an indication for nephrology referral. Rather, its potential value may lie in complementing conventional renal assessment, particularly because it is inexpensive, readily available, and routinely calculable from parameters commonly measured in patients with T2DM [33, 34]. Thus, when FIB‐4 is already available as part of the metabolic and hepatic assessment of a patient with T2DM, an elevated value may serve as an additional signal prompting clinicians to ensure comprehensive renal, hepatic, and cardiovascular assessment. The moderate discrimination and relatively low sensitivity observed in our study further support its potential use, if prospectively validated, as an adjunctive rather than a standalone marker of kidney dysfunction. This concept should also be considered within the rapidly evolving therapeutic landscape of T2DM and MASLD, in which SGLT‐2 inhibitors and GLP‐1 receptor agonists provide benefits extending beyond glycemic control, whereas dual glucose‐dependent insulinotropic polypeptide (GIP)/GLP‐1 receptor agonists and liver‐directed therapies are further expanding treatment options for selected patients [32]. Whether these interventions modify the relationship between FIB‐4 and kidney dysfunction remains unknown. In the evolving cardio–renal–metabolic framework, prospective studies should, therefore, investigate whether combining FIB‐4 with established renal markers, cardiovascular risk assessment, novel kidney injury biomarkers, or imaging‐based measures provides incremental value for identifying patients with CKD.

An apparently paradoxical finding in our study was the higher prevalence of CKD among male participants, contrasted with the lower odds of CKD in men compared with women in the multivariate analysis. This discrepancy is likely due to confounding factors related to age and cardiovascular comorbidity. Indeed, men in our cohort were older than women (mean age of 68.5 ± 10.2 vs. 58.9 ± 7.4 years, *p* = 0.047), had a higher HellenicSCORE II (6.4 ± 4.1 vs. 4.8 ± 4.0, *p* < 0.001), and were more frequently diagnosed with CVD (*p* = 0.002). In multivariate analysis, female sex remained independently associated with the presence of CKD. This observation aligns with earlier studies indicating that there is a greater occurrence of CKD among women [35, 36], whereas men appear to have a more rapid progression to ESRD [37]. Hormonal, genetic, and socioeconomic factors have been proposed to contribute to these sex‐related differences [37]. However, our findings should be interpreted with caution, as the predominance of male participants in our cohort may limit generalizability. Furthermore, the relatively small number of patients with CKD may have increased the risk of model instability and residual overfitting.

The strength of our study lies in evaluating the association between FIB‐4 and CKD in a consecutive, real‐world cohort of patients with T2DM who had not previously undergone systematic assessment of liver disease. The observed association between FIB‐4 and the presence of CKD suggests that FIB‐4 may capture clinical information related to kidney dysfunction beyond its traditional role as a liver fibrosis index. Moreover, to our knowledge, this is the first study to compare multiple noninvasive scores for their ability to detect CKD.

Nonetheless, certain limitations should be acknowledged. First, the retrospective and cross‐sectional design limits the ability to draw conclusions about the causal relationship between FIB‐4 and CKD progression. Furthermore, this was a single‐center study conducted at a specialized diabetes clinic, which may limit the generalizability of our results. However, by including consecutive, routine‐care patients who were not preselected for liver or kidney disease, we likely minimized, but did not eliminate, selection bias. Second, CKD was defined by GFR measurements over a short 3‐month period, without longitudinal kidney function trajectories. Therefore, transient renal dysfunction cannot be excluded. In addition, because our study was retrospective, data on albuminuria were unavailable. Third, 30% of patients lacked sufficient data to calculate GFR. Missing renal function data were primarily due to incomplete laboratory records in the retrospective database, rather than to clinical characteristics or selective exclusion. To minimize selection bias, we compared patients with available GFR data to those without. Although the two groups did not differ significantly in baseline characteristics, selection bias cannot be ruled out. Fourth, patients were intentionally included irrespective of MASLD status, as our objective was to investigate the association between FIB‐4 and CKD in a consecutive, routine‐care T2DM population rather than exclusively in patients with established MASLD. Nevertheless, the absence of systematic liver imaging or transient elastography prevented assessment of the prevalence and severity of MASLD and limits the mechanistic interpretation of the observed association. Specifically, we cannot determine whether elevated FIB‐4 reflects occult hepatic fibrosis or captures a combination of age‐related, metabolic, inflammatory, and CKD‐related systemic alterations. Therefore, the observed association between FIB‐4 and CKD should not be interpreted as evidence that hepatic fibrosis itself underlies this relationship. Fifth, residual confounding related to several clinical and therapeutic factors cannot be excluded. Data on albuminuria, duration and severity of hypertension, glycemic variability, inflammatory biomarkers, socioeconomic factors, and detailed longitudinal medication exposure were unavailable or incomplete because of the retrospective study design. Although treatment duration with SGLT‐2 inhibitors and GLP‐1 receptor agonists was included in our analyses, information on dosage, adherence, treatment intensity, use of angiotensin‐converting enzyme (ACE) inhibitors and angiotensin receptor blockers (ARBs), mineralocorticoid receptor antagonists, and temporal changes in therapy was not systematically available. Therefore, the potential influence of these factors on the observed associations cannot be fully accounted for. Sixth, the predominance of male participants may limit the generalizability of our findings. Finally, the HellenicSCORE II was originally developed for cardiovascular risk estimation in primary‐prevention populations, whereas our cohort included patients with already established CVD. However, in our study, the score was used exploratorily to assess its association with prevalent CKD rather than to estimate cardiovascular risk. Its performance for CKD identification should, therefore, be interpreted as hypothesis‐generating and requires validation in independent populations.

In conclusion, in a consecutive, routine‐care cohort of patients with T2DM who had not previously been evaluated for MASLD or renal disease, higher FIB‐4 values were independently associated with the presence of CKD. These findings extend previous observations in selected high‐risk subgroups and suggest that FIB‐4 may capture clinically relevant information associated with CKD, even in routine‐care T2DM populations. However, given the cross‐sectional design of the present study, no conclusions can be drawn regarding the ability of FIB‐4 to predict future renal outcomes. Prospective longitudinal studies are needed to determine whether FIB‐4 can predict the onset or progression of CKD and whether its incorporation into risk‐stratification algorithms provides additional clinical value.

## Author Contributions

K.S.: data curation, writing (original draft), and conceptualization. D.S.K.: writing (review and editing), methodology, formal analysis, and conceptualization. M.M., S.M., P.K., and P.Z.: data curation. M.P. and E.K.: methodology and investigation. T.P.: investigation and project administration. S.A.P: investigation and supervision.

## Funding

No funding was received for this manuscript.

## Disclosure

All authors have read and approved the final version of the manuscript. Dimitrios S. Karagiannakis had full access to all of the data in this study and takes complete responsibility for the integrity of the data and the accuracy of the data analysis.

## Ethics Statement

The protocol was consistent with the Helsinki Declaration for human studies, as updated in 1983, and received approval from the institution′s ethics committee. Written informed consent was obtained from all participants for the anonymous use of their data.

## Conflicts of Interest

The authors declare no conflicts of interest.

## Supporting information


**Supporting Information** Additional supporting information can be found online in the Supporting Information section. STROBE statement for cross‐sectional studies.

## Data Availability

The authors confirm that the data supporting the findings of this study are available within the article.
